# Reverse engineering a gene network using an asynchronous parallel evolution strategy

**DOI:** 10.1186/1752-0509-4-17

**Published:** 2010-03-02

**Authors:** Luke Jostins, Johannes Jaeger

**Affiliations:** 1 Laboratory for Development & Evolution, University Museum of Zoology, Department of Zoology, University of Cambridge, Cambridge, CB2 3EJ, UK; 2 Wellcome Trust Sanger Institute, Hinxton, UK; 3 EMBL/CRG Systems Biology Research Unit, Centre de Regulació Genòmica (CRG), Universitat Pompeu Fabra, Dr Aiguader 88, 08003 Barcelona, Spain

## Abstract

**Background:**

The use of reverse engineering methods to infer gene regulatory networks by fitting mathematical models to gene expression data is becoming increasingly popular and successful. However, increasing model complexity means that more powerful global optimisation techniques are required for model fitting. The parallel Lam Simulated Annealing (pLSA) algorithm has been used in such approaches, but recent research has shown that island Evolutionary Strategies can produce faster, more reliable results. However, no parallel island Evolutionary Strategy (piES) has yet been demonstrated to be effective for this task.

**Results:**

Here, we present synchronous and asynchronous versions of the piES algorithm, and apply them to a real reverse engineering problem: inferring parameters in the gap gene network. We find that the asynchronous piES exhibits very little communication overhead, and shows significant speed-up for up to 50 nodes: the piES running on 50 nodes is nearly 10 times faster than the best serial algorithm. We compare the asynchronous piES to pLSA on the same test problem, measuring the time required to reach particular levels of residual error, and show that it shows much faster convergence than pLSA across all optimisation conditions tested.

**Conclusions:**

Our results demonstrate that the piES is consistently faster and more reliable than the pLSA algorithm on this problem, and scales better with increasing numbers of nodes. In addition, the piES is especially well suited to further improvements and adaptations: Firstly, the algorithm's fast initial descent speed and high reliability make it a good candidate for being used as part of a global/local search hybrid algorithm. Secondly, it has the potential to be used as part of a hierarchical evolutionary algorithm, which takes advantage of modern multi-core computing architectures.

## Background

The driving aim of systems biology is to understand complex regulatory systems. A powerful tool for this is reverse engineering, a top-down approach in which we use data to infer parameter values for a model of an entire system. This differs from the traditional bottom-up approach of building up the larger picture through individually measured simple interactions. Many methods have been developed for reverse engineering of gene regulatory networks, most of which are based on expression data from gene expression microarrays. However, most of these approaches do not consider temporal or spatial aspects of gene expression. Examples of this are methods that infer regulatory modules from expression data across different experimental conditions [1,2], or methods based on (static) Bayesian network models [3,4]. Network models with a temporal component can be used to analyse time-series of expression data [5,6]. In contrast, microarray-based approaches are impractical for investigating spatial gene expression patterns at cellular resolution, since they are usually performed on homogenised tissue. Single-cell microarray analysis is possible, but technically difficult and highly time-consuming [7].

There are many systems for which the spatial aspects of gene expression are essential. Even in single-celled organisms, spatial localisation of regulatory factors is important [8]. Spatial aspects become absolutely central for one of the most promising applications of reverse engineering: the *in silico *reconstitution of developmental gene regulatory networks. In this context, reverse engineering is used to infer the gene networks underlying pattern formation, *i.e*. to determine which genes activate or repress which other genes at which point in time and space to yield the observed dynamics of gene expression. To achieve this, a spatio-temporal approach is absolutely required.

Here, we are going to consider a computational technique which allows the inference of explicitly spatio-temporal developmental gene networks. The approach--called the gene circuit method [9,10]--involves collecting spatial gene expression data at nuclear resolution for various stages of development. This is achieved by confocal laser scanning microscopy of embryos immunofluorescently stained against particular gene products, combined with subsequent quantification of the expression data [11]. A gene network model--consisting of a set of deterministic non-linear differential equations--is then used as a computational tool to extract regulatory information from the spatial time-series data: it is formulated such that its regulatory parameters determine whether each individual regulatory interaction is activating, repressing or absent altogether. These parameters are phenomenological in the sense that they summarise the regulatory effect of each transcription factor as a single numerical value; as such, it is not possible to measure them experimentally. Instead, they must be inferred by fitting the model to the data. We employ a global optimisation procedure to reduce the least-squares difference between the two. This technique has been successfully used to gain insights into the gene regulatory networks involved in pattern formation in the early embryo of the fruit fly *Drosophila melanogaster*. The resulting models were used to unambiguously assign specific regulatory interactions to the control of specific expression features, and enabled the quantitative analysis of expression dynamics, as well as error reduction capabilities of the system [10,12-24].

One of the main problems with this approach is that both the number of equations and the number of parameters of the model--and thus the time required to run the optimisation for fitting the model to the data--increase rapidly as more genes are considered. It is thus extremely important to design effficient global optimisation algorithms to keep up with the ever-increasing scope of systems biology; efficiency in this case means both directly increasing the speed of the algorithms, as well as allowing the algorithms to run efficiently in parallel. The most commonly used algorithm for fitting gene circuit models has been parallel Lam Simulated Annealing (pLSA) [25-27]. However, pLSA has a very high computational cost, which makes it difficult to scale to more complex problems. An alternative approach dramatically increases computational efficiency by dividing the optimisation procedure into several distinct steps, some of which can easily and efficiently be run in parallel [16]. The drawback of this approach is that it depends on problem-specific assumptions about gap gene expression, and is not easily adapted to other inference problems. An extensive review of global optimisation methods for reverse engineering regulatory networks indicated that evolutionary algorithms--and in particular Evolution Strategies (ESs)--often outperform other methods at optimisation of biological network models [28]. Such Evolutionary Strategies have been implemented in systems biology parameter estimation tools [29], and in particular an adapted ES (the island-ES) has been shown to fit a gene network model more efficiently than Simulated Annealing [19]. Up to this point, the main practical advantage of the Simulated Annealing approach for the gene network optimisation problem was that it had a proven efficient parallel algorithm, whereas no parallel version of the island-ES has been applied to this problem before. In the hope of harnessing the same parallel utility that has been successful for Simulated Annealing, we developed a parallel version of the island-ES algorithm. In addition to the previously reported advantages of evolutionary algorithms [19,28], the parallel ES algorithm is also an asynchronous algorithm, in the sense that processors send and receive communications independent of the activity of others. This eliminates waiting times, and leads to additional speed-up. In this paper we describe our synchronous and asynchronous parallelisations of the island-ES, and compare their efficiencies against pLSA on a real gene network problem.

## Methods

### The Problem: Drosophila Gap Gene Circuits

One of the few developmental systems that the reverse engineering approach has been applied to so far is segment determination in the early *Drosophila *embryo. During the first three hours of development, regulatory interactions among approximately 30 segmentation genes establish a segmental pre-pattern of gene expression that determines the positions at which morphological body segments will form. The general principles by which this occurs are well understood [30]: A number of maternal mRNAs become localised at the anterior and posterior pole of the embryo during egg development. After fertilisation, long-range spatial gradients are established by diffusion of translated proteins from their localised source. These maternal gradients then regulate expression of the zygotic gap genes in broad, overlapping domains. Maternal factors and gap genes together regulate pair-rule genes (expressed in 7 stripes), which in turn regulate segment-polarity genes, whose periodic expression in 14 stripes constitutes the segmental pre-pattern. The overall structure of this regulatory network is hierarchical: Maternal genes regulate gap genes, gap genes regulate pair-rule genes, pair-rule genes regulate segment-polarity genes but not vice versa. However, while it was once believed that each layer of the hierarchy was fully determined by the layer above it, it is now known that such vertical interactions are insufficient to account for the expression patterns of their targets [13]. To fully explain the observed patterns, we need to consider the (largely repressive) horizontal interactions between genes in the same layer as well. Here, we will focus on reverse engineering of the regulation of gap genes by maternal factors and gap-gap cross-repression (using the gene circuit method) as a test problem for our optimisation algorithm.

#### Mathematical Model

We model the gap gene network using the connectionist gene circuit formalism developed by Mjolsness *et al*. [9]. Our model corresponds precisely to gene circuit models used in earlier studies of the gap gene system [13,14,16,19-21]. The state variables of the system are protein concentrations in embryonic nuclei. Our model includes *N*_*g *_= 6 maternal and gap genes (*caudal (cad), hunchback (hb), Krüppel (Kr), knirps (kni), giant (gt) *and *tailless (tll))*, plus the maternal factor Bicoid (Bcd) as an external regulatory input. Segment determination occurs along the major, or antero-posterior (A-P), axis of the embryo, and is independent of pattern formation along other axes. Therefore, we consider *N*_nuc _nuclei laid out in a row, located between 35 and 92% along the A-P axis. Each nucleus has an index *i *∈ {1, ..., *N*_nuc_}. There are *N*_nuc _× *N*_*g *_concentration values {}, each governed by an ordinary differential equation (ODE), given by(1)

The three main terms of the equation correspond to protein production, decay and diffusion. We will discuss each of these terms separately.

The production term is equal to some fraction of the maximum production rate *R*^*a*^. This fraction is given by the sigmoid regulation-expression function(2)

which takes on values between zero and one. , which contains three terms:  is the sum of activation or repression by regulators *b*, where the contribution of each *b *on gene *a *is given by its concentration multiplied by the weight of the connection between them (); the weight (or interconnectivity) matrix *W *defines the network of gene regulatory interactions: a positive value of  represents activation of gene *a *by regulator *b*, a negative value represents repression, and a value equal or close to zero represents no interaction. The second term *m*^*a*^ represents the effect of the maternal factor Bcd (itself not regulated by gap genes) on gene *a*, where *m*_*a *_is the regulatory weight (analogous to *W *above) and  is the concentration of Bcd in nucleus *i*. The final term *h*^*a *^is a threshold parameter for the sigmoid Φ(*u*^*a*^), which represents the regulatory influence of ubiquitous maternal factors.

The diffusion term  is a standard implementation of Fickian diffusion, in which proteins diffuse between neighbouring nuclei at a rate proportional to the difference between their concentrations. Diffusion depends on the distance between nuclei which halves upon every division. *D*^*a*^(*n*) is the diffusion rate of each protein; as diffusion is proportional to the square of the distance between nuclei, *D*^*a*^(*n*) is a function of the number of cell divisions that have occurred prior to time *t *(see below). Every time the nuclei divide the distance between them halves, and the rate of diffusion changes according to *D*^*a*^(*n*) = 4*D*^*a*^(*n*-1).

The decay term *λ*^*a*^ assumes that gene products decay exponentially, with a decay rate *λ*_*a *_for gene *a*.

The half life of each protein is given by ln 2/*λ*^*a*^.

The model takes account of cell division. The lengths of interphase and mitosis occur according to a well determined schedule [31], and are modelled using three rules: During interphase, a continuous rule is applied, in which equation 1 holds. During mitosis, a second continuous rule is applied, in which the production term of equation 1 is set to zero. Finally, a discrete division rule is applied, in which *n *is incremented, and each nucleus (and hence ODE) splits into two, with the concentrations of all gene products copied from the mother nucleus to the daughter nuclei. The precise timing of the mitotic schedule is given in [14].

#### Quantitative Spatial Gene Expression Data

Quantitative expression data for segmentation genes in the early *Drosophila *embryo are from the FlyEx database, available online at: http://urchin.spbcas.ru/FlyEx [32,33]. The data set used here is identical to that used in earlier studies [19,20]. Data acquisition and quantification methods are reviewed in [11]. Here, we only provide a brief summary: Expression data are acquired using laser scanning confocal microscopy of immunofluorescently stained embryos. Each embryo is stained with antibodies against three distinct transcription factors (one of which is always the pair-rule protein Even-skipped (Eve), which is used as a standard for time classification and data registration; the other two consist of various combinations of gap proteins and other components of the segmentation gene network), and a nuclear counterstain to identify the positions of the nuclei. An automated image processing pipeline is used to extract per-nucleus concentrations and combine them into high-resolution time-series of integrated data. First, each embryo image is segmented; the position and extent of each nucleus are determined using a combination of watershed and either edge detection or thresholding algorithms, and protein concentrations are averaged for each data channel in each nucleus; this converts embryo images into tables with nuclear centroid positions and average protein concentrations per nucleus. The developmental age of each embryo is determined by visual inspection (by at least two independent researchers) based on Eve expression and embryo morphology (nuclear number and morphology, membrane progression during cellularisation). Non-specific background staining is removed; this is performed based on the observation that non-specific binding of antibodies follows a two-dimensional paraboloid distribution; such a paraboloid is fitted to non-expressing regions of an expression pattern and then subtracted by an affine transformation. The expression data are registered using an affine co-ordinate transform with the position of the Eve stripes (determined by spline- or wavelet-based methods) as reference points; this minimises embryo-to-embryo variability, reducing positional errors introduced during data integration. Finally, the images are averaged for each time-class and gene, in order to create an integrated data-set.

#### Model Fitting by Optimisation

In our reverse-engineering approach (the gene circuit method) we wish to find estimates for parameter values, which are best able to explain the data. We can frame this as an optimisation problem in which we attempt to find the set of parameter values  opt that gives the minimum value of an objective function *E*(). We define the objective function for a particular set of parameter values  as the sum of squared differences between the model output and the experimental data:(3)

where the sum is over all time classes *t*, genes *a*, and nuclei *i *for which we have data, and where(4)

is the vector of parameters to be estimated, with a length of *n *= *N*_*g*_(*N*_*g *_+ 5) = 66.

As mentioned above, optimisation for complex problems such as these is non-trivial. The system of equations is non-linear with a large number of parameters to be estimated, and the fitness landscape is multimodal. A full search is impossible, and a local search (moving downhill until it finds the lowest value of the objective function) is likely to get stuck in a local minimum. Thus, we must use a global optimisation algorithm; we shall compare the parallel Lam Simulated Annealing (pLSA) algorithm with our newly developed parallel Evolution Strategy (both synchronous and asynchronous versions).

To get a value for *E*, we need to numerically solve the ordinary differential equations in Equation 1 for a particular set of parameters and initial conditions. For all algorithms given below, we numerically solved the equations using a Bulirsch-Stoer adaptive-step-size solver scheme adapted from [34].

### Optimisation Algorithms

#### Search Space Constraints

We do not need or want to search the entire, unbounded parameter space; there are certain values that we know *a priori *that parameters should not take (negative production, diffusion or decay rates, for instance), and we do not want regulatory parameters to grow without bound along the saturated arms of the sigmoid expression-regulation function. To represent this, we may either introduce absolute criteria that prevent the optimisation algorithm from assigning out-of-bounds values, or we can produce a penalty function, which increases as the parameters move further into areas of unacceptable solutions. The penalty function may be added to the objective function (as occurs in Simulated Annealing), or it may be kept separate and handled in an algorithm-specific way (as occurs in the Evolution Strategy).

For the gene network problem, we use a penalty function for the regulatory parameters , *m*^*a *^and *h*^*a*^:(5)

where Λ is a control parameter, and  is the maximum observed intensity of gene *b *in the data. The justification for using this penalty function is that it remains 0 for(6)

*i.e*. when the absolute value of the total regulatory input is below a certain threshold, but rises steeply outside of those bounds. This allows individual parameter values to become quite large, while keeping total regulatory input within strict limits. We took Λ = 10^-4^. Based on earlier results [20], we fix *h*^*a *^parameters to a value of -2.5, which reduces the number of parameters to be determined from *n *= 66 to 62.

The production, decay and diffusion rates, *R*^*a*^, *λ*^*a *^and *D*^*a *^are given explicit limits, such that any parameter value outside these limits is considered unacceptable and rejected. The ranges are 10 <*R*^*a *^< 30, 0 <*D*^*a *^< 0.3 and 5 < ln 2/*λ*^*a *^< 20 for all *a*. These search space constraints are identical to those used in earlier studies [19,20].

#### Serial Island Evolution Strategy

The evolutionary algorithm we are using is a parallel Island (*μ,λ*)-Evolution Strategy (piES). It is a parallel version of the serial Island ES algorithm developed by Fomekong *et al*., which was successfully applied to the same gene network problem [19]. We will describe this serial algorithm first. Wherever possible, we have used the same values for optimisation parameters as used in the earlier study.

The island ES algorithm operates on *N*_isl _populations of individuals, each with a population size *λ*. Each population is initialised independently, and selection, recombination and mutation are performed only within populations. A migration operation links the populations; in the serial algorithm, the best individual from each island is copied to a population randomly selected from a uniform distribution. Migration occurs every *m *generations. We took *m *= 200 (experimental runs showed little variation within the range of 50 to 200; data not shown).

We denote the set of all possible individuals as *I*, with individuals *i *in population *p *∈ {1, ..., *N*_*isl*_} denoted by vector , *i *∈ {1, ..., *λ*}. Each individual has a parameter vector associated with it, , as given in equation 4. We define a fitness function Φ(): *I *→ ℝ, which is equal to the objective function *E*() as defined in equation 3, and use the penalty function Π(): *I *→ ℝ defined in equation 5.

Selection is performed to produce a set of *μ *offspring (here, we take *μ *= *λ*/5), according to a selection operator : *I^λ ^*→ *I^μ^*. The operator selects the top *μ *individuals of the population, according to a stochastic ranking procedure based on both the fitness function and the penalty function. This stochastic ranking method--introduced by Runarsson *et al*. [35]--uses a bubble-sort-like procedure, in which an arbitrary ranking is produced, and *λ *sweeps are performed, in which each individual in turn (starting from the top) is compared to the one directly below. If the result of the penalty function for both individuals is less than or equal to zero, the fitness values of the two are compared, and the pair is ordered accordingly. If the penalty function of the top individual is greater than zero, then there is a probability *P*_*f *_that the individuals will be ordered according to their fitness, and a probability 1-*P*_*f *_that they will be ordered according to their penalty value. This procedure is ended if there is no change in the order in any given sweep. We use *P*_*f *_= 0.45, which is the value used in [19].

Recombination is then performed on the offspring, using a recombination operator *r*_λ_: *I*^*μ *^→ *I*^*λ*^. The operator first produces *λ*-*μ *individuals as direct (asexual) copies of the selected individuals in the parent population, with the fittest *λ *(*mod μ*) individuals being represented twice if *λ *is not a multiple of *μ*.

Second, *μ *additional individuals are produced by recombination; each individual  in the parent population produces an offspring  with parameter vector  by recombination between its own parameter vector , the next fittest individual's parameter vector , and the fittest individual's parameter vector , using the equation(7)

where *λ *is the recombination factor. We have taken *λ *= 0.85 as in [19].

The *λ*-*μ *individuals that do not undergo recombination are then mutated, according to the local mutation operator *m*_{*φ, α*}_: *I *→ *I*. Mutation is performed according to a non-isotropic self-adaptive mutation rule, in the sense that each individual *i *has associated with it a step size for mutation (a mutation rate) for each parameter *j*, denoted as . This allows the step size to undergo evolution under selection, giving an adaptive step-size without having to specify a specific adaptation rule. Mutation starts with a random change to the mutation rate, given by(8)

for *i *∈ {*μ *+ 1, ..., *λ*} and *j *∈ {1, ..., *n*}, where  and  are tuning parameters. We have used φ* = 1. *N*_*i *_and *N*_*ij *_are a vector and a matrix of random values sampled from a normal distribution with zero mean and unit variance, which is generated afresh each generation.

Next, we mutate the parameters  themselves, using(9)

for *i *∈ {*μ *+ 1, ..., *λ*} and *j *∈ {1, ..., *n*}, where (0, 1) is another randomly sampled normal unit vector, generated at each generation.

Finally, we apply exponential smoothing to the step sizes, to reduce fluctuations(10)

for *i *∈ {*μ *+ 1, ..., *λ*} and *j *∈ {1, ..., *n*}, where *α *is a smoothing parameter. We have taken *α *= 0.2.  then becomes the mutation rate for the next round of mutation [19].

Every *τ *generations, the populations are checked to see if they have met the termination criterion (we take *τ *= 20); at the same time, information on descent speed (the current fittest individual and time since the program was started) is saved to a log. The algorithm has two termination conditions: it either runs for a preset number of generations, or it halts when the lowest value of the objective function remains below a particular preset amount for ρ × *τ *generations. Preliminary investigation showed that the convergence time was relatively constant across runs, so we use a constant number of generations. We ran all runs for 40000 generations, which we found to be long enough for virtually all runs to converge.

#### Parallel Island Evolution Strategy

Parallelisation of the serial island-ES (iES) relies on running each population on a separate processor. Since selection, recombination and mutation operate strictly within populations, only the migration operation, checking termination criteria and recording information for log files need to be parallelised. The simplest parallelisation of the serial iES is a synchronous parallel island-ES (piES). The algorithm is synchronous in the sense that all communication occurs simultaneously across all processes; when migration or other exchange of information is required, each processor halts until all other processes have caught up, and then all information is exchanged. The synchronous algorithm does not modify the behaviour of the serial algorithm, and is deterministic in the sense that serial and parallel runs with the same set of random seeds will produce exactly the same solution.

Migration occurs according to the following scheme: A node designated the master node generates a migration schedule, in which every population is assigned another population to migrate an individual to, and this schedule is broadcast to all nodes. The individual nodes then communicate with each other point-to-point, with each individual sending the parameter values for its highest-ranking individual to its designated receiver, and replacing its lowest ranking individual with the best individuals of the population for which it is a designated receiver.

The collection of data related to descent speed and the checking of termination criteria are performed together. Every *τ *generations, the best individual in each population is backed up, and the processors communicate between each other to find the lowest value of the objective function of any individual across all populations, which the master node records to a log file along with a time-stamp. As every process is aware of the fitness of the fittest individuals in all other populations, both termination criteria can be evaluated separately on each processor.

The disadvantage of the synchronous algorithm is that processors spend a significant amount of time idle. The asynchronous piES algorithm avoids this by having the processors communicate asynchronously; for migration and other communication, each processor sends information to a memory buffer associated with the process it is communicating with, which can then receive it at a later time (whenever it is ready to receive), avoiding waiting times.

For migration, every *m *generations each process copies its best individual to the buffer of a randomly selected population, and adds any individuals in its own buffer to its population. No individuals are added if the buffer is empty. Its buffer is then cleared. To avoid buffer sizes growing without bound, each processor will only place a maximum of 10 individuals in a given population buffer at any time before waiting for the them to be picked up (this is a rare event, and we have not observed it in practise). Logging descent information and checking the termination criterion also occurs asynchronously. Every *τ *generations each processor sends the fitness of its fittest individual to the buffer of a designated master node. Every *τ *generations, the master node collects the fittest individuals, records them to a log and checks the termination criterion; if the termination conditions are met, the master node sends out a terminate signal to the buffers of all processors. Similar to migration, we avoid buffer overflows as follows: each processor will leave a maximum of 50 messages in the master node buffer before waiting for it to read them (once again, we have never observed this in practise).

#### Parallel Lam Simulated Annealing

We use the parallel Lam Simulated Annealing (pLSA) algorithm developed by Chu *et al*. [27], running on *K *processors with one processor being arbitrarily defined as the master node. Optimisation parameters are taken from Jaeger *et al*. [14]. The algorithm is described in depth elsewhere [25-27,36]. Briefly, SA functions by defining an energy, given by the objective function *E*() plus the penalty function Π () (equations 3 and 5, respectively). During each iteration (or move) of the algorithm, the *K *processors change their parameter set , to a new state , according to an adaptive move generation strategy. They then evaluate the energy difference between the old and new states Δ*E *= *E*()-*E*(); if this is negative, the move is accepted; if not, then it is still accepted with a probability *exp*(Δ*E/T*). The temperature starts at *T*_0 _and is decreased according to the Lam schedule, which gives the fastest decrease in energy possible while maintaining the system is quasi-equilibrium [25]. The Lam schedule requires information on the mean and variance of the energy over time; a set of running estimates of these are calculated using specially designed estimators [26]. The same temperature is used across all nodes, and thus all processors must periodically pool their local statistics (every *τ *iterations) to allow the temperature schedule to be maintained [27]. This pooling of statistics allows the temperature to be lowered *K *times as fast as in the serial case.

To compensate for processes which leave the quasi-equilibrium regime (due to the increased rate of temperature decrease compared to the serial case), a mixing of states is performed every *m *iterations. The energy states of all processes are collected and redistributed according to Boltzmann probability: the probability of any given processor being assigned the state of process *i *is given by , where *E*_*i *_is the current energy of process *i*. This mixing of states allows the best results to propagate, but also allows nodes to explore higher energy solutions. It strongly resembles the selection procedure used in evolutionary algorithms.

In order to avoid the final solution being affected by the initial conditions, the algorithm performs an 'initial burn', in which each processor spends *n*_init _iterations running as normal serial SA at a constant temperature *T*_0_. After that, the algorithm needs to run for another *n*_init_/*K *iterations to calculate initial statistics.

There are two types of potential stopping conditions that could be used for the algorithm: the absolute condition, and the freeze condition. In the absolute condition, the algorithm terminates after the absolute mean value of *E *remains below a target energy for a certain period of time. In the freeze condition, the algorithm terminates after the absolute energy *E *changes by less than a certain proportion over a certain period of time. As preliminary investigations showed that the final energy and the convergence time were both very variable, we exclusively use the latter.

### Algorithm Performance Metrics

All algorithm implementations write the current value of the objective function and running times to log files at regular intervals (every 20 generations for the piESs, and every 10000 iterations for pLSA); these log files are used to calculate mean descent curves with standard errors and 95% ranges for each such interval. These curves can be used to compare the value of the objective function for different algorithms at any time during an optimisation run, and give an estimate of the variability in the algorithm's performance. We choose two target values of the objective function *E*, which correspond to good solutions (*E *= 350000; previously shown to represent an accurate approximation of the data [13,14,16,19,20]) and 'good-enough' solutions (*E *= 550000), which in most cases are sufficiently close to the global solution to be usable as starting points for local search strategies [15,19,20,24]. We calculate the mean time taken to reach each target value for all runs that have actually reached it, as well as the standard error of this mean (*σ*/(), where *σ *is the standard deviation of times-to-reach-the-target, and *N*_runs _is the number of attempts in a series of optimisation runs). From this, we can calculate confidence intervals based on the assumption of normally distributed error. In addition, we calculate the success rate for reaching a target value, defined as the proportion of runs that reached that target (95% confidence intervals on this value were also calculated, using the binomial distribution). In general, we give the inverse of the time taken, which measures the efficiency of the algorithm, whereas the success rate represents its robustness. Overall performance of an algorithm needs to take into account both of these measures.

In order to assess how effective our parallelisation of the Evolution Strategy was, we calculate relative and absolute speed-up. The relative speed-up is defined as(11)

and represents a measure of the efficiency of the parallelisation in terms of communication overhead (if the relative speed-up is equal to the number of processors, then the algorithm does not loose speed due to communication overhead). The absolute speed-up is defined as(12)

where K* is the number of islands resulting in optimal serial algorithm performance, which we found to be equal to 1 (see below). The absolute speed-up measures how effective the parallel algorithm is compared to the best serial algorithm, as opposed to merely the serial version of the parallel algorithm. Thus it takes into account both communication overhead and loss in efficiency due to having a non-optimal number of islands. Note that this is a much more stringent definition of parallel efficiency than the relative speed-up (which is the value more often given for parallel algorithms), but it is also the measure most often required for practical decision making, as it puts a value on exactly how much extra speed will be gained by moving to a parallel algorithm, assuming you are using an optimal serial algorithm.

We estimated 95% confidence intervals on absolute and relative speed-up using Fieller's theorem [37] for confidence intervals on ratios of Gaussians. Errors on ratios tend to be large, which explains the large confidence intervals around the speed-up values.

Note that in the Simulated Annealing literature, the value of the objective function is often called the 'energy', and in the Evolutionary Algorithms literature the same value is often referred to as the 'fitness'. To avoid confusion, we use 'value of the objective function' or 'objective value' instead.

#### Code Implementation

The parallel (*μ,λ*)-island-ES code is implemented in C++, and is based on code written and used by Fomekong *et al*. [19] (available at http://www.science.uva.nl/research/scs/3D-RegNet/fly_ea). Parallel communication is implemented using the Message Passing Interface (MPI). The pLSA code is implemented in C, and is based on code taken from the Supplementary Material of Jaeger *et al*. [13], (available at http://flyex.ams.sunysb.edu/lab/gaps.html). Little modification was made to the original pLSA code, other than minor alterations to allow finer-scale time-stamping in the log files for descent curves.

For both algorithms, we used a parameter scrambling procedure to give the problem different starting conditions; the pLSA algorithm reads in initial parameter values, which were randomised prior to starting each instance of the program, while the piES algorithm generates its starting conditions according to a random seed, which is itself randomised for each optimisation run.

Both implementations were compiled using the Intel C++ Compiler (ICC), and both implementations make use of the QLogic implementation of the Message Passing Interface (MPI). Data analysis was performed using the statistical programming language R [38].

Source code is available from the authors upon request.

Optimisation runs were performed on the Darwin parallel cluster of the University of Cambridge High Performance Computing Facility (HPC; http://www.hpc.cam.ac.uk).

## Results

### Analysis of the Serial Island Evolution Strategy

The performance of the serial island-ES algorithm is affected by the number of islands it uses [19]. However, this dependence has never been investigated beyond four islands. This is directly relevant for our parallelisation strategy: a perfect, synchronous parallel algorithm running on *K *nodes (in which no time is lost on communication) behaves exactly like a serial algorithm with *K *islands running on a computer *K *times as fast. We can thus calculate the theoretical limits of the algorithm's relative speed-up by dividing the speed of the serial algorithm by the number of islands *N*_isl_.

We performed 48 optimisation runs each using the serial algorithm with 1, 2, 4, 8, 20 and 50 islands. The number of individuals on every island was kept constant (125), resulting in a meta-population size of 125 × *K*. Note that our individual partitioning strategy differs from that used by [19], who kept a constant meta-population size, and decrease the population size with number of islands; the latter method may be more efficient for smaller numbers of islands, but will lead to unfeasibly small population sizes above about 10 islands.

**Figure 1:**
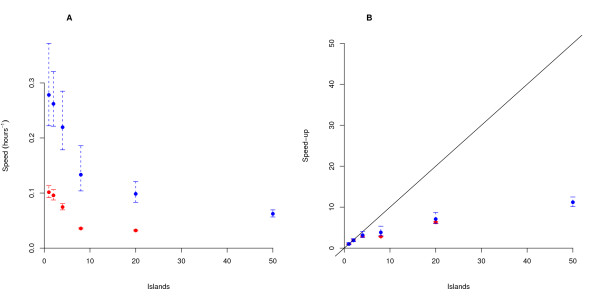
**Behaviour of the serial island-ES**. (A) The effect of the number of islands on serial algorithm performance. We plot the inverse of the time needed to reach 'good-enough' and 'good' solutions (values of the objective function less than 550000 (blue) and 350000 (red) respectively) against the number islands used. (B) Prediction of the maximum achievable absolute speed-up of a piES algorithm across different numbers of islands, calculated by assuming that each island is running on a separate processor, and there is no communication overhead. To achieve this we divide the time needed for an *N*-island ES to converge by the number of islands *N*_isl_. This measure of the optimal parallel performance is then divided with that of the fastest serial algorithm (running on 1 island). The resulting ratios are shown for 'good-enough' and 'good' solutions. The black line indicates perfect absolute speed-up. Note that no run on 50 islands was able to reach the 'good' solution value within the time limit of 36 hours for jobs on the Darwin cluster. Error bars in (A) and (B) represent 95% confidence intervals on the mean.

The amount of time required to reach both 'good-enough' and 'good' solutions is shown in Table 1, and the speed of the algorithm for various number of islands is plotted in Figure 1A. For both the 'good' and 'good-enough' targets, we observe a gradual decrease in efficiency as the number of islands increases. This decrease in efficiency occurs because the increased computational load of adding more populations out-weighs the increase in search capacity. We do not observe the dramatic increase in efficiency of 4 islands over 1 island reported by [19]; this is probably due to the different ways in which population size is partitioned in our approach.

**Table 1:** Run Times for Serial iES.

N. Islands	Time (Good-enough)	Solutions × 10^6^	Time (Good)	Solutions × 10^6^
1	3:35 (±0:54)	2.4 (±0.7)	9:51 (±1:05)	6.8 (±1.7)
2	3:49 (±0:42)	2.6 (±0.6)	10:27 (±1:01)	6.6 (±2.1)
4	4:33 (±1:03)	3.1 (±0.7)	13:24 (±1:00)	9.0 (±2.4)
8	7:30 (±2:08)	5.3 (±1.8)	28:02 (±0:47)	19.8 (±1.6)
20	10:09 (±1:52)	7.2 (±1.2)	31:17 (±0:58)	23.6 (±0.4)
50	16:03 (±1:39)	12.0 (±1.2)	*>*36:00	*>*30

The time taken to reach 'good-enough' and 'good solutions' for the serial Island ES algorithm with different numbers of islands. The number of ODE Solutions is also given. Times are given in hours and minutes (H:M), and the values in parentheses are 95% confidence intervals.

We calculated the theoretical speed of a perfect parallel algorithm on *N *nodes (assuming no communication overhead) by dividing the speed of the serial algorithm with *N*_isl _islands by *N*. From this value, we produced a theoretical absolute speed-up curve (Equation 12), by dividing each theoretical parallel speed by the highest mean speed of the serial algorithm for any number of islands. This theoretical absolute speed-up curve is shown in Figure 1B; the black line represents a perfect absolute speed-up curve, where the speed-up of an algorithm on *N *nodes is *N *times faster than the best serial algorithm. We see that a perfect piES could continue to give speed-up all the way up 50 nodes, with the ideal 50-node run being around 8-12 times faster than the best serial algorithm.

### Parallelisation Efficiency

**Figure 2:**
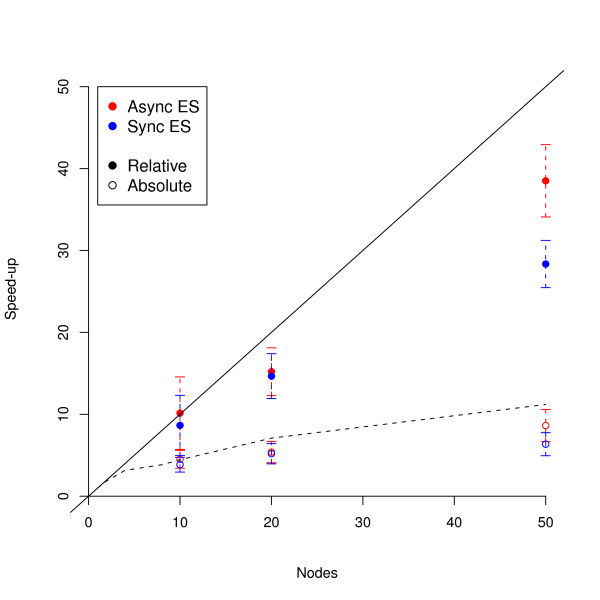
**Speed-up Curves for piES Algorithms**. The relative and absolute speed-up curves for the synchronous and asynchronous piES algorithms are shown; the solid black line corresponds to perfect speed-up, and the broken black line corresponds to the predicted maximum absolute speed-up from Figure 1B. Error bars are 95% confidence intervals on the mean.

To estimate the efficiency of our parallel algorithm, we performed 50 runs each using both synchronous and asynchronous implementations of the piES on 10, 20 and 50 processors. Running times and speed-up values are given in Table 2, and the speed-up curve is plotted in Figure 2; the speed-ups are calculated relative to the serial runs discussed above, with the absolute speed-up being calculated using the optimal island size of 1. The relative speed-up for both parallel implementations is close to perfect for 10 and 20 processors, and the efficiency of the two algorithms is largely indistinguishable. In contrast, the relative speed-up for the synchronous algorithm is low for 50 processors, while the relative speed-up remains high for the asynchronous algorithm. This indicates that communication overhead due to idle processors waiting for each other becomes significant only above 20 processors.

**Table 2:** Run Times for piES Algorithms.

Algorithm	Time (Good-enough)	Time (Good)	Relative Speed-up	Absolute Speed-up
Serial iES				
- 1 island	3:35 (±0:54)	9.51 (±1:05)	-	-
Sync piES				
- 10 nodes	0:56 (±0:07)	3:55 (±0:16)	8.7 (7.5-9.8)	3.8 (2.9-4.8)
- 20 nodes	0:41 (±0:03)	4:09 (±0:14)	14.9 (12.3-17.4)	5.2 (4.1-6.4)
- 50 nodes	0:33 (±0:03)	3:40 (±0:16)	29.2 (25.8-32.6)	6.5 (5.0-8.0)
Async piES				
- 10 nodes	0:47 (±0:06)	3:34 (±0:11)	10.3 (9.0-11.7)	4.6 (3.5-5.6)
- 20 nodes	0:40 (±0:05)	3:44 (±0:13)	15.2 (12.4-18.1)	5.4 (4.1-6.7)
- 50 nodes	0:25 (±0:02)	3:23 (±0:12)	38.5 (34.2-42.8)	8.6 (6.7-10.5)

The time taken to reach 'good-enough' and 'good solutions' for the optimal serial Island ES, and the two parallel piES algorithms, along with the relative and absolute speed-up for each parallel algorithm (comparison is between 'good-enough' solutions). Times are given in hours and minutes (H:M) and the values in parentheses are 95% confidence intervals.

The absolute speed-up (as defined in equation 12) remains significant regardless of the number of nodes used, showing that the parallel algorithm is always faster than the best serial algorithm. As expected, the absolute speed-up is generally lower than the relative speed-up: these two measures increasingly diverge as the number of processors increases, reflecting the negative effect of adding islands beyond the optimum. However, the asynchronous algorithm continues to gain speed as more nodes are added all the way up 50 nodes, with the parallel algorithm running on 50 processors nearly 10 times faster in absolute terms than the best serial algorithm.

### Comparison of Algorithms

**Figure 3:**
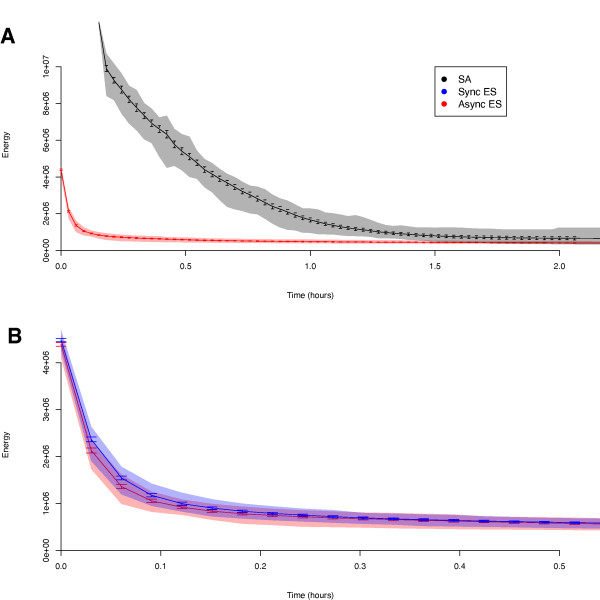
**Descent Curves for piES and pLSA Algorithms**. (A) Mean descent curves for the asynchronous piES and pLSA. (B) Mean descent curves for the asynchronous and synchronous piES. Error bars show 95% confidence intervals on the mean. Coloured regions show the area in which 95% of runs fall. All curves are for 10-node runs. Results for 20- and 50-node runs were similar (data not shown).

To compare all three algorithms (synchronous and asynchronous piES versus pLSA), we ran 50 pLSA runs each on 10, 20 and 50 processors. Example descent curves for the asynchronous piES and pLSA are shown in Figure 3A; these are the mean descent curves for the 20 processor runs (the pattern is similar for the 10 and 50 node runs). Coloured regions around the descent curves represent the region in which 95% of descent curves fall. We observe a lower initial value of the objective function, a higher initial descent speed and a lower mean convergence value for the piES compared to pLSA. In addition, the piES shows far less variation in the descent trajectory. Figure 3B compares synchronous and asynchronous piES; there is a slight increase in initial descent speed for the asynchronous algorithm over the synchronous algorithm, but both algorithms perform very similarly at later stages of the descent.

**Figure 4:**
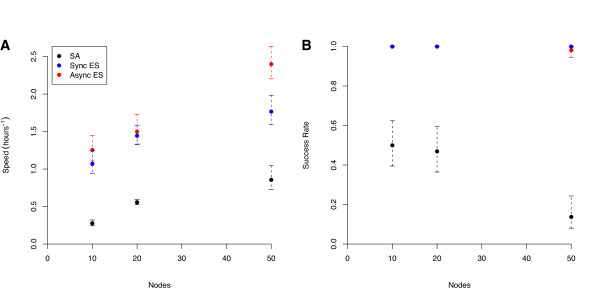
**Comparison of Algorithms for 'Good-enough' Solutions**. A comparison of (A) the speed and (B) the robustness or reliability of the three algorithms (asynchronous and synchronous piES, and pLSA) for achieving a 'good-enough' solution (an objective function value of 550000 or less). The speed is the inverse of the time, in hours, taken to achieve the target value, and the robustness or reliability is the proportion of runs that reached the target objective value. Error bars represent 95% confidence intervals on the mean.

A comparison between the times required to reach the 'good-enough' target show an approximately linear increase in speed with the number of processors for the asynchronous piES (Figure 4A). In contrast, there is a marked drop in speed-increase at 50 processors for both pLSA and the synchronous piES algorithm (Figure 4A). Both synchronous and asynchronous piES achieve virtually perfect robustness regardless of the number of processors, while pLSA becomes significantly less reliable as the number of processors increases (Figure 4B). Table 3 gives the mean number of ODE solutions performed by each algorithm to reach the 'good' and 'good-enough' solutions; as expected, the synchronous and asynchronous piES algorithms both perform roughly the same number of ODE solutions, and the relationship between number of ODE solutions for the piES and pLSA algorithms follows the same pattern as the time taken.

**Table 3:** Number of ODE Solutions for Parallel Algorithms.

Algorithm	Solutions × 10^6 ^(Good enough)	Solutions × 10^6 ^(Good)
Sync piES		
- 10 nodes	8.4 (±1.1)	34.5 (±3.7)
- 20 nodes	12.2 (±1.0)	69.8 (±5.0)
- 50 nodes	23.0 (±2.2)	149.7 (±12.1)
Async piES		
- 10 nodes	8.6 (±1.1)	37.6 (±3.4)
- 20 nodes	14.6 (±1.8)	73.7 (±5.9)
- 50 nodes	22.4 (±1.9)	168.0 (±10.1)
pLSA		
- 10 nodes	21.4 (±4.0)	26.1 (±5.3)
- 20 nodes	20.1 (±1.7)	28.8 (±5.4)
- 50 nodes	31.5 (±15.9)	43.3 (±20.7)

The mean number of ODE Solutions performed before reaching 'good-enough' and 'good' solutions for the Synchronous and Asynchronous piES algorithms, and pLSA.

**Figure 5:**
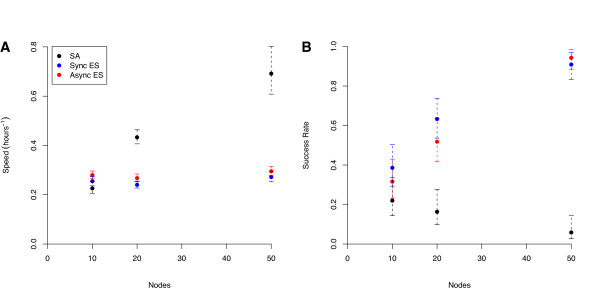
**Comparison of Algorithms for 'Good' Solutions**. A comparison of (A) the speed and (B) the robustness or reliability of the three algorithms (asynchronous and synchronous piES, and pLSA) for achieving a 'good' solution (an objective function value of 350000 or less). Axes and error bars as in Figure 4.

The behaviour of the algorithms changes significantly when they are required to reach the 'good' target (Figure 5). While the asynchronous piES remains slightly faster than the synchronous version, the speed of both algorithms is largely independent of the the number of processors. In contrast, an increase in the number of processors does increase the reliability of the algorithms, which both achieve the target nearly 100% of the time when run on 50 processors. This contrasts strongly to the behaviour of pLSA. The probability of reaching the target value falls off dramatically with the number of processors (falling as low as 4% on 50 processors); when the algorithm is made highly parallel, the vast majority of the jobs fail. However, the speed of the few runs that actually reach the target value drastically increases with the number of processors, showing that, while a majority of highly parallel runs fail, the ones that do not reach the target remarkable quickly.

## Discussion

### Parallel Efficiency of the Evolutionary Strategy

The results from the investigation of the serial algorithm show an interesting outcome: increasing the number of islands gives increased descent speed per generation, even if the number of islands is very large. In the serial case, this increased search capacity becomes outweighed by the increased computational load of the population when the number of islands increases beyond the optimal number of 1. On the other hand, it suggests that the algorithm has the capacity to be parallelised to a large number of processors.

The rate at which the serial algorithm speed decreases with the number of islands places a limit on the efficiency of the parallel algorithm (as shown by the predicted limits in Figure 1B, which were closely matched by the observed limits in Figure 2). However, this limit only applies when migration rate and per-island population size remain constant, and can likely be circumvented by tuning these values for a given set of islands or nodes. For instance, the migration rate will probably need to be elevated with an increased number of islands, to allow solutions to propagate around the larger meta-population. It may also be advantageous to decrease the population size and increase the mutation rate; a smaller population size will decrease computation time, and an elevated mutation rate will increase the search capacity of individuals. This could make solutions less stable within an island, an unwanted effect which is counteracted by the ability for good solutions to spread across the population. We have not tuned the algorithm here to allow for accurate comparison between algorithm implementations and various numbers of islands and processors. In practice, however, we can easily tune the algorithm in serial (using the predicted absolute speed-up), which will make testing these hypotheses relatively simple, especially if compared to the complex and time-consuming tuning required to reach optimal behaviour of the pLSA algorithm [39].

Both synchronous and asynchronous parallel implementations of the island-ES scale well with the number of nodes (Figure 2). That the relative speed-up is high for the synchronous algorithm is a direct result of the highly (almost embarrassingly) parallel nature of the serial algorithm, and the asynchronous algorithm improves on this even further to give a nearly perfect relative speed-up for up to 50 processors. It is worth pointing out that the change in migration schedule from the synchronous to the asynchronous implementation is relatively ad-hoc; that the algorithm does not lose efficiency despite changing its behaviour is yet another testament to the flexibility of evolutionary algorithms. Contrast this to the difficulty with which many optimisation algorithms are parallelised, including Simulated Annealing [27]. The absolute speed-up is not as striking as the relative speed-up, but it is also a far more stringent measure of algorithm efficiency. The asynchronous parallel algorithm on 10 nodes is 5 times faster than the fastest serial version, and increasing this to 50 nodes gives nearly a 10-fold increase in speed; in both cases, speed-up is significant and comparable to the predicted perfect absolute speed-up (compare Figures 1B and 2B). Algorithm performance increases yet further if we consider the robustness of convergence, which for good solutions increases drastically with the number of processors; an algorithm that has an initial descent 10-fold faster than the serial version and is almost guaranteed to converge on a good solution is indeed a powerful tool for reverse engineering biological systems.

### Simulated Annealing vs Evolutionary Strategy

The most striking feature of the descent curves of pLSA and the piES algorithm (Figure 3A) is how much faster the ES algorithm converges in almost all cases. Examining the data closely, we can see that there are three aspects that characterise the difference in the curves.

First, the piES mean descent curve begins at a much lower objective function value than that of pLSA. This is caused by differences in the initialisation procedure of the two algorithms. The pLSA algorithm begins with a single starting solution for all processors, which undergoes a high temperature 'burn period' to erase dependence on the initial condition. This is followed by a period of statistics collection (in parallel) at constant, high temperature in order to initialise estimator values for the temperature schedule. This means that each of the *K *processors has an individual initial state. In contrast, the piES algorithm starts with a much larger number of randomly created initial states equal to *λ *× *N*_isl_, allowing a far higher diversity of objective values, and thus a lower expected minimum initial objective value.

Second, the initial speed of descent is much higher for the piES. This is probably due to a particular difference in the early operation of the two algorithms. During the early stages of descent, the annealing temperature is high for pLSA, and thus there is little selection for better solutions; once the temperature is lowered the selection for better solutions increases, but simultaneously the solution is getting closer to the minimum, and the slowing associated with the decreased move size counteracts the lower temperature. The piES algorithm, in contrast, begins a full selection schedule straight away, allowing descent at maximum speed from the very start of the algorithm. Note that the reason that the piES can afford to start fast, but pLSA cannot, is that the multi-individual nature of evolutionary algorithms allows a diversity of individuals (and thus lower objective value solutions) to remain despite a decrease in mean objective value; if pLSA was to decrease at this rate, it would lose quasi-equilibrium and fail to converge, becoming stuck in a local minimum.

Third, the piES algorithm converges to a lower mean objective value across all runs than pLSA. The reason for this appears to be driven by the unreliable nature of pLSA compared to the more robust performance of the piES, as shown in Figures 4B and 5B. While pLSA can achieve very low values of the objective function (the lowest objective value for pLSA was far lower than for either implementation of the piES), the large proportion of runs that fail to reach good solutions increases the mean final objective value to above that of the piES. This also explains why we see so much faster convergence to the 'good' target in Figure 5A compared to 4A. When difficult targets are set, the failure rate for simulated annealing increases drastically, and thus the only runs that converge are in the small minority of 'good' runs. This implies that the increased speed for the 'good' target at 20 and 50 nodes is largely an artifact of the high failure rate, or at least should be judged in light of their rarity.

The unreliable nature of pLSA has been commented on before [19], with significant failure rates even for small 2-gene networks used as a test problem [27]. The fact that island-based evolutionary algorithms can be highly reliable function optimisers in large parameter estimation problems has been well established [40]. It is difficult to say precisely why our island-ES should be more reliable than a pLSA; both involve within-processor means of landscape searching (mutation/recombination in the piES, move generation in pLSA), and both of them involve a process by which solutions propagate from processor to processor (migration in the piES, mixing of states in pLSA). It is possible that the diversity within each population in the piES prevents the propagation of solutions stuck in local minima throughout the population; even if a locally minimal solution spreads to all populations, there will still be higher-objective valued individuals that will continue to search the state-space outside of this minimum, and once they achieve a lower solution than the local minimum they will begin to propagate. This does not occur in pLSA; if every processor is stuck in a local minimum, then there are no back-up individuals to allow for an escape.

### Further Methodological Improvements

The complexity of models in systems biology is constantly increasing, and thus the speed required of optimisation is always growing. The *Drosophila *segmentation system alone consists of interactions between dozens of genes and gene products [30], and to model all of them would create a drastic increase in computational complexity. Future methodological developments in optimisation will have to address how this increasing complexity will be handled.

Comparisons between gap gene circuits with 4 or 6 genes indicate that even a very moderate increase in model complexity can lead to a significant decrease in reliability of the pLSA algorithm (J. Jaeger; unpublished results). Our observations indicate that lack of robustness of the pLSA algorithm is due to the fact that most pLSA runs fail. On the other hand, those runs that converge, do so very rapidly. Therefore, efficiency of the pLSA algorithm could be improved significantly, if we managed to find a reliable method to separate failing from promising runs early on during optimisation. Such an approach has been suggested previously [41].

While the piES is both faster and more reliable than pLSA, Figure 2 shows that the communication overhead is starting to become a problem even for the asynchronous algorithm at around 50 processors. Moreover, as the number of processors increases, the difference between relative and absolute speed-up increases due to the use of populations beyond the optimum number. Therefore, it seems unlikely that the piES algorithm will scale well into the hundreds of processors and we must seek further algorithmic improvements to increase scalability, robustness and efficiency of optimisation.

One method for increasing the speed of both pLSA and piES algorithms comes from the observation that local searches tend to converge very rapidly and reliably to the global minimum, given initial conditions which are sufficiently close to the global solution [42]. This suggests a hybrid approach, which uses a global search algorithm for the initial phase--during which descent curves are steep (Figure 3)--and switches to a local search method once the descent curve has begun to flatten out. The role of the global optimisation algorithm now becomes to descend as fast as possible to a low-enough value of the objective function for the local search to converge. Many local search methods exist that can be parallelised for this purpose [43]. The critical issue is to determine the ideal switch-over point from global to local search. This can have a large impact on the efficiency of the hybrid algorithm, as a late switch wastes time on slow global descent, while a premature switch causes failure of the local search method to converge [44]. As a first step towards such a hybrid approach, local search methods have been shown to significantly improve the quality of solutions for the gap gene problem obtained by pLSA [15,24] or a serial iES [19,20]. The piES algorithm seems particularly suited for this, as it achieves a relatively low objective value very fast (Figure 3).

An alternative approach for increasing parallel efficiency--specific to evolutionary algorithms such as the piES--is the hierarchical approach [45]. A hierarchical algorithm consists of small groups of processes that run one specific aspect of an evolutionary algorithm. For example, in master-slave algorithms, a master processor performs all operations except evaluating the objective function, which is farmed out to other computers. Such master-slave clusters can in turn form part of a larger evolutionary algorithm (such as an island-ES). This becomes especially relevant as the complexity of the model, and thus the computational cost for calculating the objective function, increases. Hierarchical genetic algorithms have been applied to a variety of problems [45], and have been particularly effective in a grid computing environment [46]. Furthermore, they are ideally suited for running on multi-core architectures or highly multi-threaded graphics cards. An appropriate hierarchical implementation of the piES would be straightforward to implement, which could lead to a highly efficient, massively implementation of this parallel optimisation algorithm.

## Conclusions

Progress in systems biology crucially depends on efficient and innovative computational methods. In the case of the gap gene network, it was an innovative approach--the gene circuit method [9,10]--that allowed the extraction of regulatory information directly from the intact wild-type system. This reverse-engineering approach is generally applicable to the quantitative study of pattern-forming and other complex gene regulatory networks, if powerful optimisation methods are available to fit gene circuit models to data. Our investigation has shown that both implementations of the piES algorithm are significantly more efficient than pLSA both in terms of speed and reliability. We have demonstrated that the asynchronous piES algorithm exhibits excellent parallel speed-up for up to 50 processors, and have provided a detailed discussion of why it outperforms the pLSA algorithm on various accounts.

It was not our intention here to achieve a systematic benchmark comparison of different optimisation strategies. This has been achieved elsewhere [28,42]. Instead, we attempt to provide a practical guide on what kind of algorithm works best on a real-world biological problem, which we believe to be representative for the nature and complexity of many reverse-engineering problems which arise in the study of regulatory networks involved in physiology, development and ecology. The asynchronous piES algorithm is a powerful computational tool, which allows yet another incremental increase in the complexity of models that can be successfully fitted, and thus increases the breadth of our knowledge of the complexity of natural systems. We think that our piES algorithms not only demonstrate the power, but also the potential of evolutionary computation. Evolutionary algorithms are inspired by the processes of real-world evolution, and as such they potentially have available to them the tools that lead to the most successful optimisation run we have yet examined [47]. It is apparent that there is a whole array of modifications and improvements that can be made to such algorithms, some of which are already known, and many more that are yet to be discovered.

## Authors' contributions

LJ implemented both versions of the piES algorithm, performed optimisation runs, algorithm comparison and the statistical analysis of optimisation results. JJ proposed the research and supervised the work. LJ and JJ wrote the manuscript.
